# Chondrocytes *versus* chondroprogenitors for auricular cartilage repair: a critical comparative review

**DOI:** 10.3389/fcell.2026.1752092

**Published:** 2026-03-27

**Authors:** Ai Jiang, Wenkang Luan, Leren He

**Affiliations:** Department of Auricular Reconstruction, Plastic Surgery Hospital, Chinese Academy of Medical Sciences and Peking Union Medical College, Beijing, China

**Keywords:** auricular cartilage, cartilage stem/progenitor cells, chondrocytes, chondroprogenitors, ear reconstruction, tissue engineering

## Abstract

**Background:**

Auricular cartilage defects, whether congenital or acquired, present a significant reconstructive challenge. Autologous chondrocytes implantation remains the clinical gold standard but is limited by donor-site morbidity, limited cell availability, and dedifferentiation during expansion. Cartilage stem/progenitor cells (CSPCs) have emerged as a promising alternative, yet their comparative properties remain underexplored.

**Methods:**

A comprehensive literature review was conducted across PubMed, MEDLINE, Cochrane, and Web of Science databases up to 2026, utilizing keywords such as chondrocytes, chondroprogenitors, Cartilage stem/progenitor cells, CSPCs, auricular cartilage, ear reconstruction and tissue engineering.

**Results:**

Chondrocytes exhibit robust matrix synthesis but suffer from dedifferentiation and limited proliferation. CSPCs demonstrate superior self-renewal and immunomodulatory properties, yet face challenges of hypertrophic differentiation and inferior tissue elasticity. Co-culture and scaffold-based strategies have been employed to enhance the functionality of both cell types in engineered constructs.

**Conclusion:**

Both chondrocytes and CSPCs present distinct advantages and limitations for auricular cartilage repair. Future strategies should aim to combine their strengths, such as the functional specificity of chondrocytes and the expandability of CSPCs, to develop more effective and clinically feasible tissue-engineered constructs for ear reconstruction.

## Introduction

Auricular cartilage is a type of elastic cartilaginous tissue possessing a unique structure (Giardini-Rosa et al., 2014). Ear cartilage defects resulting from trauma, tumor resection and congenital malformations like microtia can lead to deformity, hearing loss and psychosocial issues, significantly impairing patients’ quality of life (Subaşı et al., 2014; Otto et al., 2015). In clinical practice, the repair of auricular cartilage defects has long been a major challenge in ear reconstruction due to the limited regenerative capacity of auricular cartilage (Posniak et al., 2022). Currently, autologous chondrocyte implantation is widely regarded as the gold standard for repairing cartilage defects (Pereira et al., 2016), while this method is plagued by donor-site morbidity and surgical complications (Park et al., 2017). Allogeneic cartilage transplantation carries the risk of immune rejection (Kazimierczak and Przekora, 2022), and synthetic materials are prone to causing inflammation, displacement and foreign body reactions with suboptimal long-term outcomes (Lipovka et al., 2021). Thus, the exploration of safer and more effective approaches for auricular reconstruction has emerged as a key research focus in the field of regenerative medicine.

The advent of cartilage tissue engineering has provided a new paradigm for reconstructing cartilage defects (Lee et al., 2007). Its core lies in leveraging the synergistic effects of seed cells, biomaterial scaffolds, and bioactive factors to construct cartilaginous tissue with biomimetic structure and function (Diekman et al., 2010). Despite significant progress, a fundamental question remains unresolved in cartilage tissue engineering. What is the primary bottleneck limiting clinical translation, the inadequate proliferative capacity of chondrocytes or the phenotypic instability of expandable progenitors? Chondrocytes, the gold-standard functional cells, possess superior phenotypic stability and matrix synthesis capacity but are hindered by limited self-renewal and rapid dedifferentiation during *in vitro* expansion (Vinod et al., 2018; Chen et al., 2019). Conversely, cartilage stem/progenitor cells (CSPCs) exhibit robust proliferative potential and multipotency, yet face challenges in achieving stable chondrogenic commitment and may produce tissue with inferior mechanical properties (Jayasuriya et al., 2016; Liu et al., 2023).

This debate persists despite decades of research primarily because proliferative capacity and phenotypic stability represent an inherent biological dichotomy. Studies often employ different species, cell sources and culture conditions, leading to conflicting conclusions. Consequently, the field lacks a unified consensus on which cell type offers the superior net therapeutic advantage. Therefore, this review aims to systematically compare the biological properties of chondrocytes and CSPCs, critically evaluating their respective advantages and limitations to identify strategies that might reconcile this dichotomy and pave the way for more effective, translationally relevant auricular reconstruction.

## Etiology of auricular cartilage defects and seed cells

### Structural and functional properties of auricular cartilage

**FIGURE 1:**
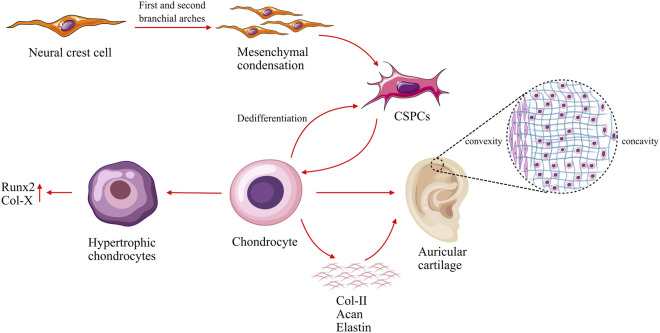
Schematic illustration of auricular cartilage development and trilaminar architecture. Cranial neural crest cells migrate to the first and second branchial arches, where they undergo mesenchymal condensation. Within these condensates, the mesenchymal cells act as CSPCs. Then CSPCs differentiate into mature chondrocytes and secrete cartilage-specific ECM components such as Col-II, Acan and elastin, which constitute the fundamental components of the auricular cartilage. Chondrocytes can undergo dedifferentiation into CSPCs or progress toward hypertrophy during *in vitro* culture. The mature auricular cartilage displays a trilaminar structure [based on Kaňa et al. (2019)]: a central zone rich in elastic fibers flanked by two peripheral layers with fewer elastic fibers. The concave outer layer contains fewer oval-shaped chondrocytes, while the convex outer layer contains abundant elongated, fusiform chondrocytes. CSPCs, cartilage stem/progenitor cells; ECM, extracellular matrix; Col-II, Type II Collagen; Acan, Aggrecan.

Histologically, auricular cartilage is composed of chondrocytes embedded within a substantial extracellular matrix (ECM) and is devoid of blood vessels, nerves, and lymphatic vessels (Su et al., 2025). Adult auricular cartilage features a unique trilaminar architecture: a central zone rich in elastic fibers, flanked by two peripheral layers with fewer elastic fibers (Kaňa et al., 2019). The typical trilaminar architecture of adult auricular cartilage is illustrated in Figure 1, and classic staining assays by Kaňa et al. have well characterized its histological features, including the differential distribution of elastic fibers and chondrocyte morphologies across the three layers (Kaňa et al., 2019). This structural heterogeneity is thought to reflect the distinct mechanical demands across the auricle cartilage as an intelligent biological material (Kaňa et al., 2019). The ECM of auricular cartilage is primarily composed of collagen, elastic fibers, and proteoglycans (Su et al., 2025). Collagen, predominantly type II, forms the fundamental fibrous network of cartilage, providing the tissue with structural integrity and functional traits (Su et al., 2025). Elastic fibers, as the defining characteristic of elastic cartilage, form an extensive and interwoven network that confers exceptional elasticity and flexibility, distinguishing it from hyaline and fibrocartilage (Visscher et al., 2021; Su et al., 2025). Proteoglycans, particularly aggrecan (Acan), consist of numerous glycosaminoglycans chains covalently linked to a core protein and bear negatively charged groups like carboxyl or sulfate (Cohen et al., 1998; Mow and Guo, 2002; Wei and Dai, 2021), a molecular architecture that enables them to attract water ions and thereby influence the permeability and mechanical properties of cartilage (Mow and Guo, 2002; Wiggenhauser et al., 2017). With its complex structure, auricular cartilage possesses high mechanical strength to maintain its shape (Otto et al., 2015; Chang et al., 2018). The notable capacity of auricular cartilage to withstand deformation rather than bearing pressure is largely attributable to the synergistic roles of ECM components, which is optimized for flexibility and resilience (Su et al., 2025).

### Etiologies of auricular cartilage defects and current therapeutic strategies

The etiologies of auricular cartilage defects are complex, and can be broadly categorized into two groups: congenital and acquired factors. Microtia, one of the most prevalent congenital malformations, manifests as partial or complete auricular underdevelopment, exhibits an incidence of roughly 0.8–17.4/10,000 (Luquetti et al., 2012; Luan et al., 2025), and frequently co-occurs with atresia of the external auditory canal and malformations of the middle ear (Wahdini et al., 2024). The pathogenesis of microtia has not been fully elucidated, and it is believed to be associated with genetic factors, environmental factors, or vascular disruption during embryonic development (Wilkes et al., 2014). Acquired factors are also prevalent, primarily encompassing trauma, tumor resection, and iatrogenic injury (Lei and Huang, 2022).

The current strategies for cartilage reconstruction comprise autologous cartilage grafts, allogeneic transplants, synthetic implants, and tissue engineering techniques (Ma et al., 2017; Yi et al., 2019; Nedunchezian et al., 2022). The clinical gold standard for auricular reconstruction is autologous cartilage grafting (Gates-Tanzer et al., 2025), most notably utilizing rib cartilage to sculpt a stable framework for the correction of microtia (Chen et al., 2022). However, this procedure is hampered by significant drawbacks, prominently donor-site morbidity and the subsequent resorption and deformation of the implanted graft (Liu et al., 2023). While allogeneic cartilage and synthetic materials eliminate the risk of donor site injury, the inherent immunogenicity means that immune rejection and disease transmission hazards persist (Kazimierczak and Przekora, 2022). Synthetic grafts can even lead to scaffold exposure, occurring at a significantly higher rate than autologous materials (Zhao et al., 2009). Representing a breakthrough in addressing immune rejection, cartilage tissue engineering introduces a novel approach to auricular cartilage reconstruction by using autologous cells, which should be recognized as a promising strategy for auricle repair (Liu et al., 2023).

### The pivotal role of seed cells

Seed cells play a central role in cartilage tissue engineering, as they are critical determinants of the quality of engineered cartilage grafts and subsequent therapeutic outcome (Shi et al., 2017). The properties for ideal seed cells are shown as follows: 1) minimally invasive sourcing with easy access and high yield; 2) strong growth potential with fast proliferation and active metabolism; 3) low immunogenicity and inflammation risks; 4) integrating well with native tissue; 5) excellent mechanics and long-term stability (Su et al., 2025). Chondrocytes and CSPCs represent promising cell sources for tissue engineering as they satisfy most outlined criteria. Autologous chondrocytes are the sole seed cells to have been utilized in clinical practice (Shi et al., 2017). However, the dedifferentiation and consequent phenotypic loss of chondrocytes during *in vitro* amplification are issues that cannot be overlooked (Zhang et al., 2009). CSPCs provide a promising alternative, given their robust capacities for proliferation and differentiation (Otto et al., 2022), but tissues derived exclusively from stem cells may lack the requisite elasticity (Cohen et al., 2018). The synergistic application of chondrocytes and CSPCs offers a promising pathway for translating experimental studies into clinical practice. For instance, a co-culture study by Luan et al. demonstrated that CSPCs can activate chondrocytes via paracrine interleukin-6 (IL-6), thereby synergistically enhancing the proliferation, migration, and chondrogenic capacity of both cell types (Luan et al., 2025).

## Biological properties of chondrocytes and clinical applications

### Origins and acquisition

Auricular cartilage is derived from cranial neural crest cells, which migrate to the first and second branchial arches and undergo mesenchymal condensation (Anthwal and Thompson, 2016). Within these condensations, the mesenchymal cells act as chondroprogenitor cells, which then differentiate into mature chondrocytes responsible for secreting the elastic-specific ECM that defines the structural and mechanical properties of the pinna (Figure 1) (Anthwal and Thompson, 2016). This developmental process is tightly regulated by an evolutionarily conserved transcriptional network and key signaling pathways such as BMP and fibroblast growth factor (FGF) (Anthwal and Thompson, 2016). Chondrocytes within the mature auricle remain in a quiescent state (Yanaga et al., 2012), with only minimal synthesis of ECM components like type II collagen (Col-II) and Acan to maintain tissue integrity, as vascular scarcity limits nutrient supply and metabolic activity, complicating regeneration and repair processes (Su et al., 2025).

Given the limited regenerative potential of cartilage (Posniak et al., 2022), autologous grafts from the rib, nasal septum, and auricle represent ideal options (Lin and Lawson, 2007; He et al., 2018). A comparative analysis by He et al. revealed that nasal septal chondrocytes exhibited the highest proliferation rate, whereas auricular chondrocytes yielded the greatest total cell number, highlighting their respective efficiencies as cell sources (He et al., 2018). Costal chondrocytes have strong proliferation and chondrogenic capacity, but as hyaline cartilage, costal cartilage differs from auricular elastic cartilage in matrix composition and mechanical properties, requiring phenotypic focus during *in vitro* induction and *in vivo* regeneration (He et al., 2018). For patients with congenital microtia, the residual auricular cartilage constitutes a valuable autologous cell source. The auricular cartilage from microtia patients demonstrated striking similarities to normal cartilage in its structure and biochemistry, featuring standard ECM compositions and chondrocyte phenotypes as evidenced by the expression of Sox9, Col-II, and elastin (Melgarejo-Ramírez et al., 2016). However, our previous studies have demonstrated that compared to normal auricular cartilage, microtia cartilage exhibited a disorganized histoarchitecture with a significant reduction in IL-6 expression and an aberrant presence of tissue stem cells (Luan et al., 2025). Another study also found that microtia chondrocytes *in vitro* exhibited a slower rate of dedifferentiation, reduced expression of stem cell-related genes, weaker migratory capacity, and inferior cartilage regeneration potential *in vivo* compared to healthy counterparts (Gu et al., 2018). Further study by Yanaga et al. revealed that exposure to FGF-2 was able to stimulate both proliferation and hyaluronic acid secretion of microtia-derived chondrocytes (Yanaga et al., 2012). Collectively, these results indicate that future strategies should aim to re-awaken this compromised functionality of microtia chondrocytes through targeted molecular or environmental interventions.

### Characteristics of *in vitro* culture

**FIGURE 2:**
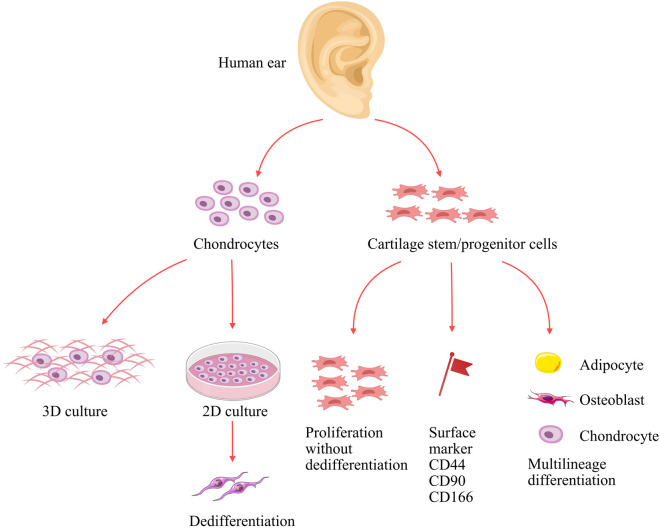
Schematic review of the biological properties of chondrocytes and CSPCs in auricular cartilage. CSPCs, cartilage stem/progenitor cells.

Chondrocytes are typically expanded *in vitro* using a two-dimensional monolayer culture system, which supports rapid proliferation. It has been reported that infant chondrocytes exhibit a higher growth rate than adult chondrocytes in monolayer culture, indicating their superior proliferative capacity (Mortazavi et al., 2017). Nevertheless, upon *in vitro* expansion, chondrocytes undergo dedifferentiation (Figure 2), losing their characteristic morphology and biological functions (Wu et al., 2025). The dedifferentiation process, marked by a morphological change from polygonal to fibroblast-like spindle shapes and an aberrant matrix composition (decreased Col-II and increased type I collagen, Col-I), results in a significant loss of cartilage elasticity and mechanical strength (Liu et al., 2023). Further investigation revealed that increased calcium influx is closely associated with early chondrocyte dedifferentiation (Wu et al., 2025). This influx downregulates MYC mRNA expression, which in turn reduces the levels of key transcription factors Sox5/Sox6, leading to decreased ECM synthesis and ultimately initiating dedifferentiation (Wu et al., 2025). Additionally, the TGF-β/Smad3 signaling pathway is critical for maintaining the chondrocyte phenotype. Studies showed that salidroside can activate this pathway to promote chondrocyte proliferation, enhance cartilage ECM synthesis and downregulate Col-I, thereby inhibiting dedifferentiation (Sun et al., 2020).

Given the limitations of monolayer culture, three-dimensional (3D) systems are extensively used for the *in vitro* culture and phenotypic preservation of chondrocytes (Klimek et al., 2022). He et al. revealed that the 3D chondrogenic system effectively induced dedifferentiated chondrocytes to regain functional phenotype, enabling the regeneration of mature cartilage (He et al., 2020). Established 3D culture models encompass pellet (Watts et al., 2013), scaffold-based culture (Chen et al., 2014), as well as scaffold-free systems such as cartilage sheets (Hayashi et al., 2017) and microtissues (Shajib et al., 2023). The pellet culture represents a stable, biomaterial-free approach and is widely regarded as the gold standard for studying chondrocyte redifferentiation and mesenchymal stem cells (MSCs) differentiation *in vitro* (Watts et al., 2013). A variety of biological and synthetic scaffolds are utilized in cartilage tissue engineering, and the critical characteristics of these scaffolds include mechanical strength, biocompatibility, biodegradability, porosity, and absence of toxicity (Bichara et al., 2012; Chen et al., 2014). Cartilage sheet technology is a scaffold-free strategy wherein chondrocytes are cultured to over-confluence, forming a dense, ECM-rich sheet that aids in the preservation of the native cellular phenotype. Cartilage sheets derived from infant chondrocytes exceeded those from adult cells in terms of thickness and ECM richness, evidencing their enhanced chondrogenic potential (Mortazavi et al., 2017). Cartilage microtissues, assembled from either chondrocytes or bone marrow stromal cells (BMSCs) using a customized microwell-mesh platform, rapidly accumulate a homogeneous cartilage-like ECM, positioning them as highly promising building blocks for cartilage defect repair (Shajib et al., 2023).

### Application of chondrocytes in the repair of cartilage defects

Cartilage defects exceeding 2 cm^2^ are currently managed via transplanting autologous culture-expanded chondrocytes, and as the resident cell in cartilage, chondrocytes serve as the prime candidate for repairing cartilage defects (Rikkers et al., 2021). A key challenge in auricular engineering lies in acquiring an adequate quantity of chondrogenic cells for the fabrication of a cartilaginous framework (Bichara et al., 2012). Based on comprehensive literature review, an estimated 100–150 million cells are needed to engineer an adult human ear-shaped cartilage, with the exact number depending critically on the type and porosity of the scaffold material employed (Bichara et al., 2012), presenting a fundamental barrier for microtia patients who have minimal ear cartilage available for cell harvesting (Liu et al., 2023). This substantial cell demand necessitates consideration of not only cell sourcing but also the profound effects of high cell density on phenotypic stability. For mature chondrocytes, high seeding density (mimicking the physiological density of native auricular cartilage) acts as a potent barrier against dedifferentiation *in vitro*, preserving the expression of Col-II and Acan while downregulating Col-I levels (Cao et al., 2020; He et al., 2020). For CSPCs, high-density culture conditions, such as those employed in pellet culture systems, have been shown to promote chondrogenic differentiation, as evidenced by upregulation of Sox9 and formation of cartilage-specific matrix (Otto et al., 2022; Gardner et al., 2023). However, it is noteworthy that such densities in large-scale constructs may introduce diffusion limitations, highlighting the need for optimized porosity to balance phenotypic stability and metabolic viability (Posniak et al., 2022).

Currently, research utilizing human chondrocytes for auricular engineering has made significant progress through various approaches. Ting et al. first combined fibrin gel with human costal chondrocytes to generate 3D ear-shaped cartilage in nude mice, but the constructs suffered significant volume loss and shape deformation over an 8-week *in vivo* period (Ting et al., 1998). In a subsequent study, Haisch et al. focused on assessing the feasibility of generating ear-shaped cartilage using human nasoseptal chondrocytes harvested from clinical biopsies, and they confirmed the implants exhibited excellent shape retention post-implantation, with only mild contraction observed (Haisch et al., 2002). Pomerantseva et al. demonstrated that extensively expanded autologous auricular chondrocytes could form stable ear-shaped cartilage in an immunocompetent sheep model when seeded onto porous collagen scaffolds. The resulting neocartilage exhibited robust expression of glycosaminoglycans, Col-II and elastin, and the overall ear shape was preserved with minimal shrinkage (<10%) after 20 weeks *in vivo* (Pomerantseva et al., 2016). Bernstein et al. further showed that late-passage human auricular chondrocytes (up to passage 5) still retained their chondrogenic capacity, suggesting a strategy to overcome limited cell availability (Bernstein et al., 2018).

A recent study by Zhang et al. elucidated a novel mechanism in which an upregulated desmin-integrin β1-MAPK axis in auricular chondrocytes potentiated chondrogenic differentiation and thereby enhances the mechanical strength of the ECM (Zhang et al., 2023). These finding identifies promising targets for cell selection and manipulation in elastic cartilage engineering. Collectively, these advances in culture optimization, scaffold engineering, and mechanistic understanding are progressively transforming chondrocytes into a clinically viable cell source for auricular reconstruction.

## Biological properties of CSPCs and clinical applications

### Sources and acquisition

CSPCs are a kind of stem cells identified in animal and human auricular cartilage, possessing the capacity to differentiate into chondrocytes (Liu et al., 2023). The presence of CSPCs has been documented in both healthy and microtia ear cartilage (Otto et al., 2022). According to our earlier work, normal auricular cartilage is primarily composed of mature chondrocytes, accounting for 85.36%, and the content of CSPCs is only 4.39% (Luan et al., 2025). In contrast, microtia tissue contains a large number of CSPCs with a proportion as high as 57.50%, while chondrocytes only account for 40.62% (Luan et al., 2025). This study suggests that the pathogenesis of microtia may involve impaired differentiation of CSPCs into functional chondrocytes, leading to a dysregulated microenvironment in auricular cartilage. The auricular perichondrium also serves as an important source of CSPCs. Kobayashi et al. were the first to report the existence of stem cells within the auricular perichondrium (Kobayashi et al., 2011). Under specific culture conditions, these cells demonstrate efficient chondrogenic differentiation and are capable of forming engineered cartilage tissue with functional elasticity and mechanical strength (Oba et al., 2022). They also exhibit favorable characteristics including minimal donor site morbidity and high proliferative capacity (Oba et al., 2022). Studies by Xue et al. have shown that the proliferation rates of CSPCs derived from the perichondrium are higher than that of those from auricular cartilage (Xue et al., 2016), providing valuable guidance for selecting ideal seed cells for auricular cartilage regeneration.

The isolation methods of CSPCs are mainly based on their biological characteristics such as selective adhesion ability. The classical fibronectin-based differential adhesion method enriches CSPCs by seeding a digested cell suspension onto fibronectin-coated dishes, followed by a brief incubation and then removal of non-adherent cells (Xue et al., 2016). Studies have confirmed that CSPCs isolated via this method exhibit robust proliferative capacity without losing their multipotent differentiation capacity (Otto et al., 2022). Recent investigations have introduced laminin as an alternative substrate to fibronectin in differential adhesion assays where CSPCs isolated using laminin possess higher proliferative potential and stronger osteogenic/adipogenic ability, but lower Col-II expression (Vinod et al., 2021), implying that specific ECM proteins can enrich for functionally distinct stem cell subpopulations.

### Characteristics of *in vitro* culture

CSPCs exhibit distinctive growth kinetics during *in vitro* culture. Compared with mature chondrocytes, CSPCs demonstrate superior proliferative potential (Gu et al., 2022). In a comparative study of CSPCs, BMSCs and chondrocytes, it was found that the proliferative capacity of CSPCs and BMSCs was significantly higher than that of chondrocytes, and the DNA content of CSPCs was also higher than that of the other two groups after 28 days of culture (Gu et al., 2022). In another study on platelet-rich plasma (PRP) scaffold-based cartilage regeneration, CSPCs demonstrated superior migration and proliferation capabilities compared to chondrocytes, alongside a heightened chondrogenic propensity relative to BMSCs, collectively highlighting their advantages over both cell types (Wang et al., 2019). The work by Rikkers et al. further established that CSPCs maintain their stemness and chondrogenic potential even after multiple passages, enabling large-scale expansion for potential clinical applications (Rikkers et al., 2021).

CSPCs are also characterized by strong self-renewal capacity, assessed by colony-forming unit assays where single cells proliferate into large colonies (Xue et al., 2016). While there is no consensus on the markers for CSPCs, they are typically defined according to the International Society for Cell Therapy (ISCT) criteria for MSCs, being positive for CD73, CD90, and CD105, and negative for CD14, CD34, CD45, and HLA-DR (Liu et al., 2021). Numerous studies have documented the association of certain markers (e.g., CD44 and CD90) with CSPCs (Liu et al., 2023). The specific expression of these molecules provides a reliable basis for the identification and purification of CSPCs. For example, a subsequent investigation has demonstrated that CD90, CD44 and CD166 are the primary surface markers for CSPCs (Luan et al., 2025). This marker profile can be used to distinguish them from primary chondrocytes, which predominantly express mature chondrocyte markers such as Col-II, Sox9, and Acan (Luan et al., 2025).

### Multilineage differentiation and regulation

Chondrogenic differentiation represents the most crucial characteristic of CSPCs and serves as the foundation for their application in cartilage repair. Chondrogenesis *in vitro* is commonly achieved through pellet culture, supplemented with chondrogenic induction medium including dexamethasone, ascorbic acid, sodium pyruvate, insulin-transferrin-sodium selenite, and key chondrogenic growth factors such as TGF-β1 or TGF-β3 (Zhang S. et al., 2019; Otto et al., 2022). The synergistic effect of these factors leads to the upregulation of key chondrogenic markers (Col-II, Sox9, and Acan) and the deposition of a characteristic ECM (Col-II and GAGs), resulting in the formation of neocartilage (Otto et al., 2022). Gardner et al. revealed that BMP9 can induce CSPCs to form larger-volume pellets and enhance the ordered arrangement of collagen fibers, which is particularly important for the repair of tissues requiring specific mechanical properties such as auricular cartilage (Gardner et al., 2023).

The osteogenic and adipogenic differentiation potential of CSPCs remains a critical indicator for verifying their stemness and may play a role in the repair of osteochondral composite defects. Osteogenic differentiation is typically induced using a medium containing dexamethasone, β-glycerophosphate, and ascorbic acid (Otto et al., 2022). Following induction, cells form mineralized nodules, a hallmark of osteogenesis (Otto et al., 2022). However, the osteogenic potential of CSPCs is weaker than that of BMSCs. Under specific induction conditions, they may even fail to form a discernible mineralized matrix, which corresponds to their physiological role in cartilage repair rather than bone formation (Rikkers et al., 2021). Adipogenic differentiation is induced using a medium containing dexamethasone, isobutyl methylxanthine, indomethacin, and insulin, and successful induction is characterized by the intracellular accumulation of lipid droplets (Tangchitphisut et al., 2016; Otto et al., 2022). Notably, CSPCs isolated using different methods may exhibit varied osteogenic and adipogenic potential. As previously described, CSPCs enriched through laminin-based selection exhibit superior osteogenic and adipogenic capacities compared to those isolated by fibronectin (Vinod et al., 2021).

### Application of CSPCs in the repair of cartilage defects

Currently, the application of CSPCs for the repair of auricular cartilage defects remains confined to preclinical studies, yet accumulating evidence highlights their therapeutic potential. A systematic review by Liu et al. identified CSPCs and perichondrial progenitor cells (PPCs) as promising cell sources for auricular reconstruction, noting their superior chondrogenic differentiation potential compared to adipose-derived stem cells and BMSCs, as well as the advantage of *in situ* harvest from microtia tissue without additional incisions (Liu et al., 2023). Oba et al. demonstrated that micro 3D spheroids derived from human auricular perichondrial chondroprogenitors gave rise to stable cartilage within 8 weeks and the neocartilage contained elastic fibers that was histologically comparable to its native auricular cartilage (Oba et al., 2022). Similarly, Derks et al. isolated porcine PPCs and demonstrated their robust chondrogenic differentiation potential *in vitro*, highlighting their promise as a cell source for cartilage tissue engineering in large-animal models (Derks et al., 2013).

Residual auricular cartilage from microtia patients represents an ideal autologous source of CSPCs. Otto et al. characterized fibronectin-adhering progenitor cells from human adult, pediatric and microtia auricular cartilage, demonstrating that these cells exhibit robust proliferative capacity without loss of multipotent differentiation potential (Otto et al., 2022). The accessibility of ear chondroprogenitor cells, which can be obtained through non-deforming biopsy of the healthy ear or from microtia remnants, makes them a key solution to a persistent challenge in auricular cartilage tissue engineering (Otto et al., 2022). Notably, Togo et al. showed that CSPCs not only matched BMSCs in adipogenic and osteogenic differentiation, but more importantly, outperformed them *in vivo* cartilage reconstruction, reinforcing the therapeutic promise of CSPCs for auricular repair (Togo et al., 2006).

Despite these promising results, several challenges remain before clinical translation. While stem cell-based auricular reconstruction has shown success in small animal models, transplantation in large animals is still lacking, and long-term safety and efficacy studies are needed. Additionally, standardized protocols for CSPCs isolation, expansion, and quality control must be established to ensure reproducible outcomes.

## Comparative analysis of chondrocytes and CSPCs

### Cell sources and acquisition

As summarized in Table 1, chondrocytes are predominantly isolated from mature cartilage tissue, such as costal, nasal septal and auricular cartilage (He et al., 2018). In a standard procedure, a small cartilage specimen is surgically obtained and subjected to enzymatic digestion to yield a single-cell suspension (Luan et al., 2025). However, this approach is associated with several significant limitations such as donor-site injury (Liu et al., 2023), limited tissue availability especially in children or patients with low cartilage reserves, and constrained cell yield due to the low density and age-dependent decline in chondrocyte viability and function (Cavalli et al., 2019). By comparison, CSPCs can be isolated from a wider range of sources, including cartilage itself as well as surrounding tissues like the perichondrium and synovium, offering greater flexibility in procurement (Xue et al., 2016). Representing an ideal choice for auricular cartilage reconstruction, CSPCs have been successfully isolated from porcine auricular cartilage and perichondrium, and confirmed to express typical MSCs markers (CD29, CD44, CD90) with trilineage differentiation potential (Xue et al., 2016). While perichondrium-derived CSPCs are associated with enhanced proliferation (Xue et al., 2016), chondroprogenitors from the auricular cartilage are linked to superior chondrogenic capacity *in vivo* (Zhang X. et al., 2019). The synovium is also a reservoir of CSPCs. Notably, synovium-derived stem cells (SDSCs) demonstrate an elevated chondrogenic differentiation capacity, evidenced by their significantly higher expression of chondrogenic markers (Col-II and Acan) compared to BMSCs and chondrocytes *in vitro* (Kubosch et al., 2017). Beyond direct isolation from cartilage and adjacent tissues, CSPCs can also be acquired from the dedifferentiation of mature chondrocytes. The process of *in vitro* monolayer expansion induces chondrocyte dedifferentiation, characterized by a loss of the chondrogenic phenotype and a concomitant acquisition of stem cell-like properties, constituting a source of CSPCs under certain conditions (Vinod et al., 2018).

**TABLE 1:** Comparative framework of chondrocytes and CSPCs for auricular cartilage repair.

Characteristics	Chondrocytes	CSPCs
Proliferation capacity	Limited; slow expansion rate; undergo senescence after limited passages; difficult to obtain sufficient cell numbers for large-scale reconstruction (Bichara et al., 2012; Cavalli et al., 2019; Liu et al., 2023).	Superior; rapid self-renewal; maintain high proliferative potential even after multiple passages; suitable for large-scale expansion (Xue et al., 2016; Rikkers et al., 2021).
Phenotypic stability	Prone to dedifferentiation in monolayer culture (loss of Col-II, increase in Col-I); require 3D systems to maintain phenotype or redifferentiate (He et al., 2020; Liu et al., 2023).	Maintain multipotency; risk of hypertrophic differentiation rather than dedifferentiation (Kachroo and Vinod, 2020; Luan et al., 2025).
Cell source and availability	Limited; derived from mature cartilage (auricle, rib, nasal septum); donor-site morbidity; low yield, especially in microtia patients (He et al., 2018; Liu et al., 2023)	Diverse; isolated from cartilage, perichondrium, synovium and dedifferentiated chondrocytes; higher accessibility (Xue et al., 2016; Vinod et al., 2018).
Differentiation capacity	Committed to chondrogenic lineage; negligible osteogenic/adipogenic potential; produce cartilage-specific ECM (Col-II and Aggrecan) (Chen et al., 2019; Luan et al., 2025).	Multipotent; capable of chondrogenic, osteogenic, and adipogenic differentiation; robust chondrogenic output under proper induction (Gu et al., 2022; Luan et al., 2025).
Immunogenicity	Low in steady state (only express MHC-I); but MHC-II can be upregulated under inflammatory conditions, increasing rejection risk (Huey et al., 2012; Osiecka-Iwan et al., 2018).	Very low; minimal MHC-I, no MHC-II or co-stimulatory molecules; maintain low immunogenicity even under inflammatory stimulation (El Haddad et al., 2011; Lee et al., 2019).
Inflammatory response	Highly sensitive; inflammatory cytokines (IL-1β or TNF-α) impair ECM synthesis and induce catabolism (Richardson and Dodge, 2000).	More resilient; maintain viability and reparative function in inflammatory microenvironments; modulate matrix remodeling (Jiang et al., 2015).

Abbreviations: CSPCs, cartilage stem/progenitor cells; Col-II, type II collagen; Col-I, type I collagen; 3D, three-dimensional; ECM, extracellular matrix; MHC, major histocompatibility complex; IL-1β, interleukin-1β; TNF-α, tumor necrosis factor-α.

### Differentiation capacity and phenotypic stability

As terminally differentiated cells, chondrocytes are primarily committed to maintaining a mature phenotype and synthesizing cartilage-specific ECM. However, their phenotypic stability is poor, rendering them highly susceptible to dedifferentiation during *in vitro* monolayer culture or upon serial passaging (Vinod et al., 2018). Even in 3D culture, chondrocytes subjected to multiple passages exhibit compromised differentiation capacity along with a reduction in the quality of the synthesized ECM (Wang et al., 2013). In contrast, the primary risk for CSPCs *in vitro* is their tendency toward hypertrophic differentiation. However, studies have demonstrated that CSPCs isolated via the fibronectin differential adhesion assay express significantly lower levels of the hypertrophy markers Runt-related transcription factor 2 and type X collagen compared to non-adherent cells and freshly isolated chondrocytes (Kachroo and Vinod, 2020).

Simultaneously, chondrocytes exhibit a highly restricted differentiation potential, which is largely confined to the chondrogenic lineage with negligible potential for osteogenic or adipogenic differentiation (Luan et al., 2025). This limited plasticity constrains their application in the repair of complex tissues. By comparison, CSPCs represent classic mesenchymal multipotency, capable of differentiating into chondrogenic, osteogenic, and adipogenic lineages under specific induction (Luan et al., 2025). Research by Xue et al. established that CSPCs isolated from auricular cartilage and perichondrium can form mineralized nodules in osteogenic induction medium and lipid droplets in adipogenic induction medium (Xue et al., 2016). Another study by Gu et al. has proposed that under same induction conditions, the cartilaginous tissue formed by CSPCs is superior to that by chondrocytes in terms of quality, quantity, and mechanical properties (Gu et al., 2022). Specifically, after isolating porcine CSPCs and culturing them in agarose gel for 28 days, their gene expression levels related to cartilage synthesis, as well as the contents of GAGs and collagen, were significantly higher than those in the chondrocyte group, suggesting CSPCs possess greater promise as seed cells in tissue engineering (Gu et al., 2022). Although the multipotency of CSPCs provides a distinct advantage for the regeneration of complex tissues like osteochondral defects, it also introduces the risk of undesirable ectopic tissue formation, necessitating precise control over culture conditions and inductive signals to mitigate this outcome.

### Major histocompatibility complex (MHC) expression and immunogenicity

Immunogenicity is primarily determined by the expression of MHC molecules on the cell surface (Liu et al., 2022). Accumulating evidence supports the immunoprivileged nature of cartilaginous tissues, which elicit minimal to no detectable immune response following implantation (Huey et al., 2012). The precise mechanisms require further elucidation. A study has confirmed that human chondrocytes express MHC-I molecules but not MHC-II, CD80 or CD86, collectively contributing to their inherent low immunogenicity (Huey et al., 2012). However, this low immunogenic state can be subverted under inflammatory conditions. Stimulation of chondrocytes with inflammatory factors such as interferon-γ (IFN-γ) induces the upregulation of MHC-II molecule expression, which in turn elevates the risk of recognition and attack by the host immune system and ultimately triggers an immune rejection response (Osiecka-Iwan et al., 2018).

CSPCs enriched by fibronectin differential adhesion can be categorized as MSCs as they comply with the identification criteria established by the ISCT (Vinod et al., 2018). They exhibit low expression of MHC-I markers, and no expression of MHC-II and costimulatory molecules, thus allowing for immune surveillance evasion (El Haddad et al., 2011). Studies have indicated that CSPCs maintain low levels of MHC-II expression even under IFN-γ stimulation, which was comparable to MSCs and further underscores their dual advantages in immune privilege and immunomodulation (Lee et al., 2019).

### Inflammatory response

Chondrocytes are highly sensitive to the inflammatory microenvironment. Inflammatory cytokines like interleukin-1β (IL-1β) and tumor necrosis factor-α (TNF-α) induce catabolic-anabolic dysregulation in chondrocytes, impairing ECM synthesis and enhancing MMP-mediated degradation (Richardson and Dodge, 2000). Prolonged exposure to an inflammatory microenvironment has been shown to induce marked functional impairment and even apoptosis in chondrocytes (Liu-Bryan and Terkeltaub, 2015). In contrast, CSPCs exhibit greater resilience to inflammatory microenvironment. Jiang et al. provided clear evidence that CSPCs maintain viability even in IL-1β-induced inflammatory conditions and counteract cartilage damage via a mechanism involving the regulation of protease/inhibitor balance and matrix remodeling (Jiang et al., 2015). It has also been suggested that the reparative function of CSPCs in an inflammatory environment can be mediated by mechanical stress via the Hippo-YAP signaling pathway, whose activation effectively promotes cell proliferation and chondrogenesis (Yao et al., 2022).

## Conclusion and prospects

Cartilage tissue engineering provides a promising approach for auricular cartilage defect repair, with seed cell selection being the core determinant of success. This review systematically compares chondrocytes and CSPCs, highlighting their distinct strengths and limitations: chondrocytes excel in cartilage-specific matrix synthesis but suffer from limited proliferation and dedifferentiation risks, while CSPCs offer robust self-renewal and low immunogenicity yet face challenges of hypertrophic differentiation and inferior tissue elasticity.

To advance clinical translation, future studies should focus on three testable directions: 1) optimizing chondrocyte-CSPCs co-culture strategy to integrate their complementary advantages and mitigate phenotypic instability; 2) developing biomimetic scaffolds with tailored porosity and elastic modulus, combined with growth factor immobilization such as BMP9 and FGF-2, to enhance large-scale construct maturation under high cell density; 3) targeting key signaling pathways (calcium-MYC for chondrocytes and Hippo-YAP for CSPCs) to improve *in vitro* expansion efficiency and phenotypic stability. Additionally, standardized cell procurement protocols and long-term clinical trials are essential to bridge the gap between basic research and clinical application, ultimately delivering safe, effective reconstructive solutions for patients with auricular defects.

## Limitations

Despite advances, it must be acknowledged that the current literature on auricular cartilage repair remains relatively limited compared to other cartilage types. Most studies are confined to *in vitro* experiments or small animal models, with a paucity of large animal studies and clinical trials. This reflects the niche but clinically important nature of the field, and underscores the need for further preclinical and clinical investigation to validate these promising findings.
